# Transcriptomic basis underlying the evolution of female horn plasticity in scarab beetles

**DOI:** 10.1186/s12983-026-00597-3

**Published:** 2026-02-05

**Authors:** Cuicui Qi, Wenqing Zhang, Yonggang Hu

**Affiliations:** State Key Laboratory of Resource Insects, Southwest University, Chongqing, 400715 China

**Keywords:** Phenotypic plasticity, Beetle horns, Sexual dimorphism, Exaggerated traits, Nutrition-sensitive growth

## Abstract

**Background:**

Exaggerated traits in insects often evolve through nutrition-sensitive growth, yet studies have focused largely on male exaggeration widespread in nature, leaving the developmental and evolutionary basis of female exaggeration and the extent to which plasticity differs between sexes poorly understood.

**Results:**

Here, we investigated the transcriptomic basis of female horn plasticity in three *Onthophagus* beetles that differ in horn morphology and nutritional responsiveness. Phylogenetic analyses indicate that rudimentary female horns represent the ancestral condition, with exaggerated, nutrition-sensitive horns evolving in derived lineages. Comparative transcriptomics reveal that enhanced female horn plasticity arises primarily through the recruitment and modulation of conserved regulatory networks, with limited contributions from taxon-restricted genes. Despite the shared reliance on conserved modules in two species with enhanced female horn plasticity, the transcriptional repertoires underlying horn plasticity are largely lineage-specific. In *O. rectecornutus*, where both sexes bear exaggerated horns, sex comparisons demonstrate that males and females rely on the expression of largely distinct gene sets, with only a small fraction of shared nutrition-responsive genes.

**Conclusions:**

Together, our results show that exaggerated female horns arise through a mosaic of conserved and lineage-specific regulatory mechanisms, with strong sex bias in transcriptional control. More generally, our findings highlight how conserved developmental toolkits can be rewired in lineage- and sex-specific ways to evolve novel, plastic, and exaggerated traits.

**Supplementary Information:**

The online version contains supplementary material available at 10.1186/s12983-026-00597-3.

## Background

Scaling relationships between organs and body size (static allometries) are a fundamental feature of insect morphology [1, 2]. These relationships are not fixed but exhibit remarkable plasticity in response to environmental factors, particularly nutritional regime, thereby linking developmental physiology with evolutionary change [3, 4]. Variation in nutritional availability modifies growth rates and developmental timing to alter both overall body size and relative organ proportions [5–13]. Mechanistically, nutrition acts largely through conserved insulin/insulin-like growth factors (IGFs) and TOR signaling pathways, and by modulating endocrine factors such as ecdysone and juvenile hormone, thereby coupling metabolic state to tissue-specific growth responses [14–16]. Because different organs vary in their sensitivity to these cues, nutritional plasticity can generate the allometric diversity observed within and across species. Such nutrition-dependent effects on growth not only structure morphological variations but also provide developmental substrates for evolutionary diversification. For instance, interspecific variation in horned dung beetles spans exaggerated, reduced, or secondarily lost weapons [17]; evolutionary shifts in trait scaling generate caste-specific worker and soldier morphologies in *Pheidole* ants [18]; and intraspecific divergence in wing size of brown planthopper *Nilaparvata lugens* reflects adaptive shifts in nutritional and dispersal strategies [19, 20]. Additional striking examples include repeated evolution of enlarged male mandibles in broad-horned flour beetles [21] and stag beetles [22], where exaggerated traits enhance fitness under variable nutritional pressures.

The horned beetle genus *Onthophagus* is one of the most species-rich animal groups, with more than 2000 species formally described [23]. Horns in *Onthophagus* beetles, representing a textbook example of an evolutionary innovation and sexually dimorphic trait, function mainly as male weapons in contests for mates [3, 24–26]. Horn scaling has diversified extensively across populations and species, which spans modest to strong positive allometry and even polyphenic expression (for instance in *O. taurus*), where males adopt either a small, nearly hornless sneaker form or a large, fully horned fighter form depending on body size thresholds [27]. Studies of *Onthophagus* beetles provide important insights into the developmental regulation and evolution of horn scaling. Highly sigmoidal horn allometry in *O. taurus* depends on the co-option of several distinct signaling pathways, including deeply conserved insulin/IGF-like pathway (e.g., *Insulin Receptor*, *FoxO*), sex-determination pathway (e.g., *doublesex*), Hedgehog (Hh) signaling pathway (e.g., *Hh*, *smoothened*, *patched*), and serotonin pathway[28–34]. Recent study further revealed that exaggerated nutritional sensitivity in male horns evolved through the amplification and redeployment of ancestrally plastic gene networks, while lineages that secondarily lost morphological responsiveness show a dramatic reduction in gene expression plasticity [17]. However, most research on scaling and nutrition-dependent plasticity in *Onthophagus* and other insects has concentrated on exaggerated male traits, whereas females remain largely overlooked, despite the fact that they too exhibit substantial morphological and physiological variations. It is therefore unclear whether males and females rely on shared developmental mechanisms to regulate scaling, or whether sex-specific regulatory architectures underlie their divergent evolutionary trajectories.

While most *Onthophagus* females exhibit only rudimentary or no horns, notable exceptions offer unique opportunities. In *O. sagittarius*, sexual dimorphism is reversed: females possess prominent head horns, whereas males have secondarily lost them. In *O. rectecornutus*, both sexes display exaggerated head horns. Together, these species provide an exceptional comparative framework to explore the development and evolution of female horn scaling and compare the underlying trajectories of exaggerated horns between sexes. Here, we investigated transcriptomic basis of female horn plasticity in three *Onthophagus* species with contrasting horn morphologies and nutritional responsiveness. We first mapped the phylogenetic history of female horn expression, then compared transcriptomic responses to nutrition in horn primordia across species. We identified shared and lineage-specific transcriptional repertoires, evaluated the relative contributions of conserved versus taxon-restricted genes (TRGs) across species, and finally compared the transcriptomic landscape underlying the exaggerated horns between sexes in *O. rectecornutus*. Our study provides new insights into the transcriptional architecture of female horn plasticity and its role in the diversification of exaggerated traits.

### Methods

#### Animal husbandry

All the beetle species used in the study were collected from Fujian, China, and maintained under laboratory conditions as previously described [35]. Low-nutrient (hay-based) and high-nutrient (grass-based) cow dung were obtained from local organic farm during winter and summer, respectively, which is followed by homogenization to ensure nutrition uniformity. To facilitate experimental manipulation and observation, larvae at approximately the second instar were transferred to artificial brood balls prepared in 12-well plates filled with dung mixture. All beetles were reared under identical environmental conditions throughout development.

### Scaling relationship measurements

Thoracic width was used as the proxy of body size to assess horn scaling relationships as described previously [35] (Supplementary Figure S1A). Because horn shape and position vary among the three focal species, measurements were standardized accordingly. In *O. rectecornutus*, horn length was measured linearly along the outer contour from the base to the tip (Supplementary Figure S1B, C). In *O. bivertex* and *O. sagittarius*, horn length was defined as the vertical height from the head capsule to the horn tip (Supplementary Figure S1D, E). Images were captured with a stereomicroscope (Leica S9i, Singapore) and measured using ImageJ (v.1.51j8, NIH).

### Sample preparation and total RNA extraction

The relative position of the pupal compound eye during the prepupal stage was used as a morphological landmark to define equivalent developmental stages across species [36, 37]. Horn primordia were dissected from late prepupae (Supplementary Figure S1F) of both sexes under low- and high-nutrition conditions in all three species and subjected to RNA isolation. For each treatment, a minimum of three independent biological replicates were collected. In brief, pupal head was dissected out from the larval cuticle in phosphate-buffered saline (PBS) solution. The epidermal region between the dorsal compound eyes where the horn primordia located was cut off and the adhering muscle and fat body were carefully removed. After homogenization of the dissected samples on ice with an electric grinder, total RNA was extracted using the RNeasy Plus Micro kit (Cat. No. 74034, Qiagen, Germany). The RNA amount and purity of each sample was quantified using NanoDrop 2000c (Thermo Fisher Scientific, USA) and the RNA integrity was assessed by Bioanalyzer 2100 (Agilent, CA, USA) (Supplementary Table S6).

### Transcriptome sequencing and analysis

Total RNA samples were shipped to LC-Bio Technology Company Limited (Hangzhou, China) where they were normalized and sequenced on an Illumina NovaSeq™ 6000 platform. Raw reads were processed using Trimmomatic (v.0.39) [38] to remove adapters and low-quality sequences. For species with available genomes, reads were aligned to the genome using HISAT2 (v.2.2.1) [39] and gene expression quantified with StringTie (v.2.2.1) [40]. For species without a reference genome, transcriptomes were assembled de novo using Trinity (v.2.15.2) [41], transcripts were then annotated using Trinotate (v.4.0.2) [42], and gene expression was quantified by aligning reads back to the assembled transcriptome using Bowtie2 (v.2.5.4) [43] and estimating abundances with RSEM (v.1.3.3) [44].

Differentially expressed genes (DEGs) were identified by comparing horn primordia dissected from individuals reared under low- and high-nutrition conditions using DESeq2 (v.1.34.0) [45] with an absolute log_2_ fold change (log_2_FC) > 1 and a *P* value < 0.05. These DEGs were classified as nutrition-responsive genes (NRGs). The Gene Ontology (GO) [46] and Kyoto Encyclopedia of Genes and Genomes (KEGG) [47] analyses were performed to identify enriched functional categories. Orthologous genes shared among the three focal species were identified using reciprocal BLAST [48] searches to enable cross-species comparisons.

### Analysis of nutrition-dependent expression trends of NRGs across species and sexes

To investigate expression trends of NRGs across species and sexes, log₂FC between low- and high-nutrition conditions were calculated for each gene, capturing both the magnitude and direction of nutritional response. Genes were then classified according to their observed expression trends across species and sexes (e.g., concordant upregulation, concordant downregulation, or divergent responses). For each trend category, the number of genes exhibiting that pattern was quantified. Exact binomial tests were used to evaluate whether the observed counts deviated from an expected distribution (H₀: p = 0.50 for two-category comparisons or H₀: p = 0.33 for three-category comparisons). In addition, Fisher’s exact tests were applied to evaluate enrichment patterns among species- and sex-shared NRGs.

For visualization, average expression profiles were calculated for all genes within each expression-trend category to determine their positions in the line plots under low- and high-nutrition conditions. To quantify expression variability, coefficients of variation (CVs) were calculated for each gene using trimmed mean of M values (TMM)-normalized expression values within each species. CV was defined as the ratio of the standard deviation to the mean expression level. CVs were computed separately for each nutritional condition within each species, and statistical comparisons within and across groups were conducted using Welch’s *t*-tests with Bonferroni correction. Differences were considered statistically significant when adjusted *P* values (*Padj*) were < 0.05 following Bonferroni correction.

### Identification of TRGs

TRGs were identified by querying protein sequence against the NCBI non-redundant (Nr) database [49] using BLASTp (v.2.16.0) [50] with an E-value cutoff ≤ 1. Proteins lacking homologous matches were classified as taxon-restricted. To reduce false negatives due to limited genomic representation of *Onthophagus* in public databases, candidate TRGs were further validated through reciprocal BLAST searches across the three focal *Onthophagus* genomes or transcriptomes. This two-tiered approach ensured robust identification of TRGs.

### Phylogenetic analyses

To reconstruct evolutionary relationship among dung beetles, we analyzed four species from two genera within the subfamily Scarabaeinae: *Digitonthophagus gazella*, *O. sagittarius, O. rectecornutus,* and *O. bivertex*. Phylogenetic inference was performed in IQ-TREE using the Maximum Likelihood method based on protein sequences. Protein datasets for *D. gazella* (GCA_036711955.1) and *O. sagittarius* (GCA_036711965.1) were obtained from the NCBI [51]. For *O. bivertex* and *O. rectecornutus*, protein sequences were predicted from de novo transcriptome assemblies.

### Statistical analysis and image processing

Exact binomial tests were used to test whether observed proportions of gene categories deviated from expected values in R (v.4.1.3) [52]. This approach was applied to species-specific plasticity genes, up- and down-regulated NRGs. Fisher’s exact test was applied to evaluate the shared NRGs in R. Transcriptomic visualizations were generated in R. NRG counts were visualized in GraphPad Prism (v.8.0.2, GraphPad Inc., San Diego, CA, USA, 2023), and figures were assembled using Adobe Photoshop CS6 (Adobe Inc., San Jose, CA, USA, 2012).

## Results

### Evolution of nutrition-responsiveness of head horns in female scarab beetles

**Fig. 1:**
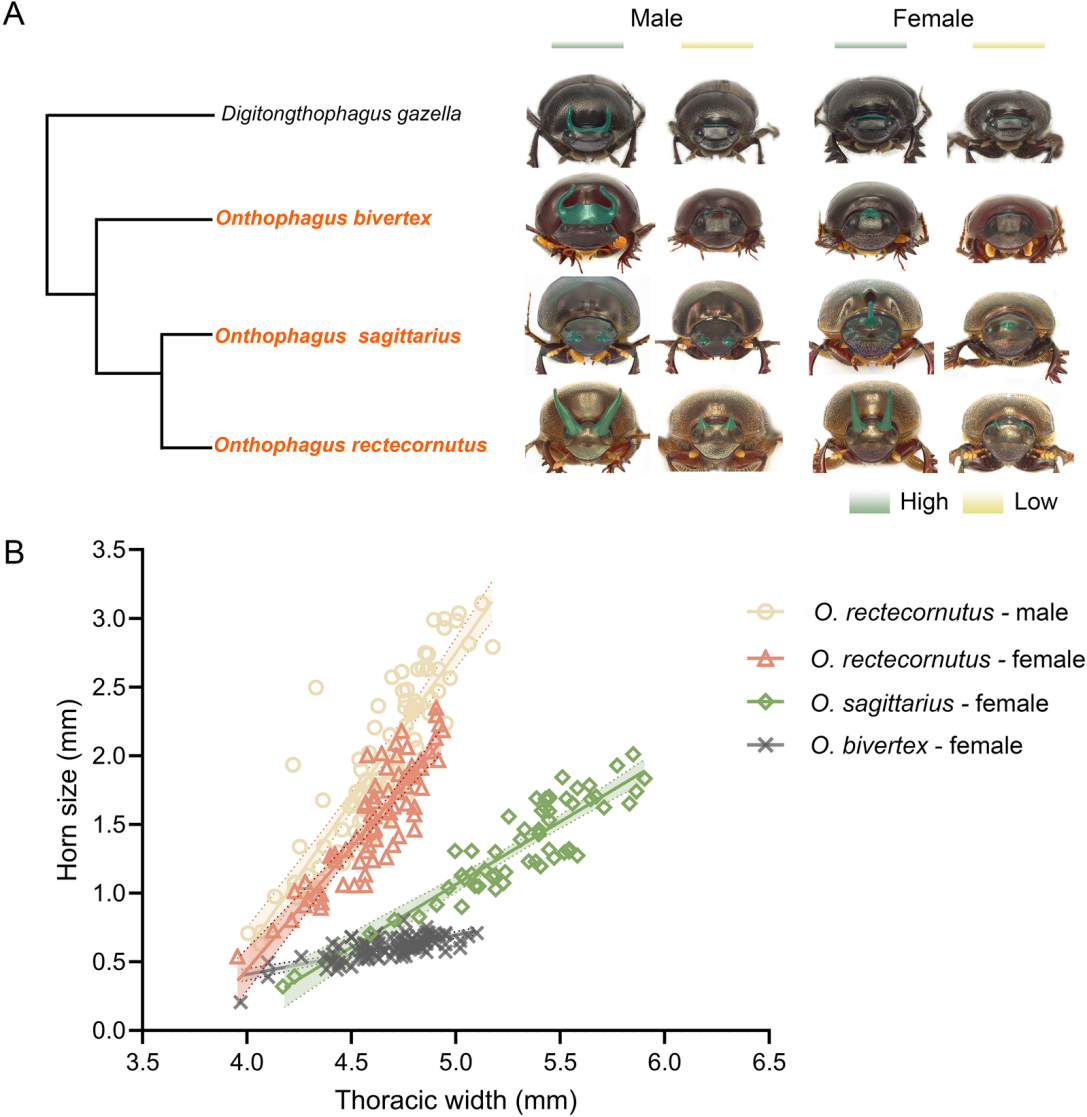
Allometric evolution of nutrition-dependent growth of head horns in scarab beetles. A: Phylogenetic tree for four scarab beetle species, with the three focal *Onthophagus* species highlighted in orange. Morphology of head horns indicates nutrition- plastic growth under high- (green) and low-nutrient (yellow) conditions. Dorsal-posterior head horns and their corresponding regions were highlighted with a green pseudocolor to emphasize their morphology. B: Allometric relationships between head horn size and body size for *O. rectecornutus*, *O. sagittarius*, and *O. bivertex*. Solid lines represent fitted allometric slopes and shaded areas indicate 95% confidence intervals

We focused on three *Onthophagus* species that differ strikingly in the nutritional sensitivity of female head horns. (1) *O. bivertex*, in which females bear minute, mild nutrition-sensitive posterior head horn—a morphology commonly seen across the genus; (2) *O. sagittarius*, whose females develop a single posteromedial head horn that shows moderate nutritional responsiveness; and (3) *O. rectecornutus*, in which females produce a pair of bilateral posterior head horns with pronounced responsiveness to nutrition (Fig. 1A, B). To trace the evolutionary history of this trait, we reconstructed a phylogeny using genome-wide single-copy orthologs. Phylogenetic mapping indicated that the ancestral condition of female is hornless or minimally horned, as exemplified in *D. gazella* and *O. bivertex*, while the evolution of enhanced nutritional responsiveness in *O. sagittarius* and *O. rectecornutus* reflects a derived state (Fig. 1A).

### Enhancing nutritional plasticity partially depends on the plastic expression of genes

Gene-expression changes underlying morphological plasticity do not necessarily track the magnitude or direction of the plastic trait itself [53]. However, a recent study on male head horns has shown that enhanced nutritional plasticity is primarily driven by an increase in the number of nutrition-responsive genes [17]. To test whether this pattern also applies to females, we performed RNA-seq on head horn primordia from females at the prepupal stage reared under low- and high-nutrition conditions in the three focal species (Supplementary Figure S2). We identified DEGs as those exhibiting an absolute log₂FC > 1 and *P* < 0.05. These genes were subsequently defined as NRGs.

**Fig. 2:**
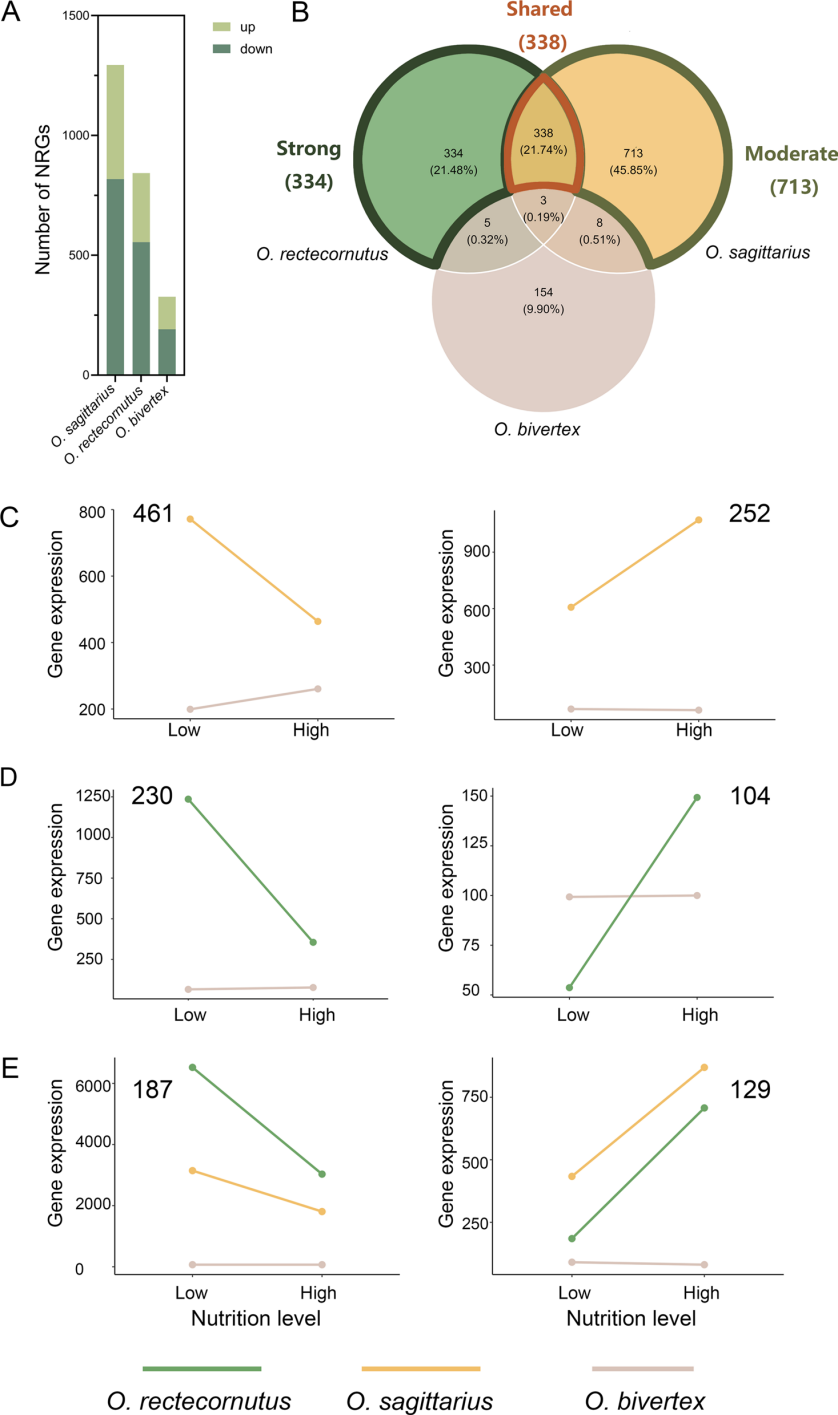
Recruitment of nutrition-irresponsive genes into plastic regulatory networks. A: Counts of NRGs that are up-regulated (light green) or down-regulated (green) in each species. B: Distribution of orthologous NRGs species specific to or shared in *O. rectecornutus*, *O. sagittarius*, and *O. bivertex.* C–E: Evolutionary divergence of NRGs expression pattern across three gene sets: moderate NRGs (C), strong NRGs (D), and shared NRGs (E). For C–E, log₂FC values between low- and high-nutrition conditions were calculated for each gene to determine direction of nutritional response, and genes were subsequently classified into different categories. Numbers indicate the number of genes within each category, and line plots represent average expression profiles of all genes within each defined category

Using these criteria, we revealed 843 NRGs in *O. rectecornutus*, 1294 in *O. sagittarius* and 327 in *O. bivertex*, respectively (Fig. 2A; Supplementary Figure S3). The two derived species (*O. sagittarius* and *O. rectecornutus*), which show more elaborate nutritional plasticity in horn morphology, exhibited more NRGs than *O. bivertex*. However, nutrition-responsive horn plasticity is not tightly correlated with the extent of gene expression plasticity: *O. sagittarius*, with moderate horn plasticity, had more NRGs than *O. rectecornutus*, in which female horns exhibit strong nutritional responsiveness. This result indicates that more elaborate horn plasticity is not simply explained by NRGs abundance, but likely reflects additional regulatory mechanisms.

### Shared and lineage-specific transcriptional repertoires underlying female horn plasticity

To assess whether female head horns rely on shared transcriptional repertoires in response to nutrition, we compared NRGs between low- and high-nutrition treatments across the three focal species. Within the subset of common putative orthologs, only three genes responded consistently across all species. By contrast, 338 NRGs (Fisher’s exact test, *P* < 0.001) were shared between *O. sagittarius* and *O. rectecornutus*, but not with *O. bivertex* (Fig. 2B). Because female head horns in *O. sagittarius* and *O. rectecornutus* exhibit enhanced nutritional sensitivity, whereas those of *O. bivertex* respond only weakly, we interpret these 338 genes as shared NRGs. This pattern suggests that the evolution of heightened nutritional plasticity in female horn morphology was driven less by modification of ancestral mild plastic responses than by the recruitment of nutrition-insensitive gene expression programs into a plastic regulatory framework.

We further identified 713 (H₀: p = 0.5, *P* < 0.001) and 334 (H₀: p = 0.5, *P* = 0.67) species-specific NRGs in *O. sagittarius* and *O. rectecornutus*, respectively. Given their moderate and strong nutrition-dependent horn growth in *O. sagittarius* and *O. rectecornutus*, these sets are considered as moderate and strong NRGs, respectively, highlighting both common and lineage-specific mechanisms underlying plastic horn development. Expression dynamics analyses revealed a consistent bias toward downregulation under high-nutrition conditions compared with low-nutrition conditions: among moderate NRGs, 461 (H₀: p = 0.5, *P* < 0.001) were downregulated and 252 (H₀: p = 0.5, *P* < 0.001) upregulated (Fig. 2C; Supplementary Table S1); among strong NRGs, 230 (H₀: p = 0.5, *P* < 0.001) were downregulated and 104 (H₀: p = 0.5, *P* < 0.001) upregulated (Fig. 2D; Supplementary Table S1); and among shared NRGs, 187 were downregulated and 129 upregulated (Fig. 2E; Supplementary Table S1). This consistent pattern suggests that the evolution of female horn plasticity involved not only the activation of new transcriptional programs but also the systematic suppression of ancestral expression modules.

**Fig. 3:**
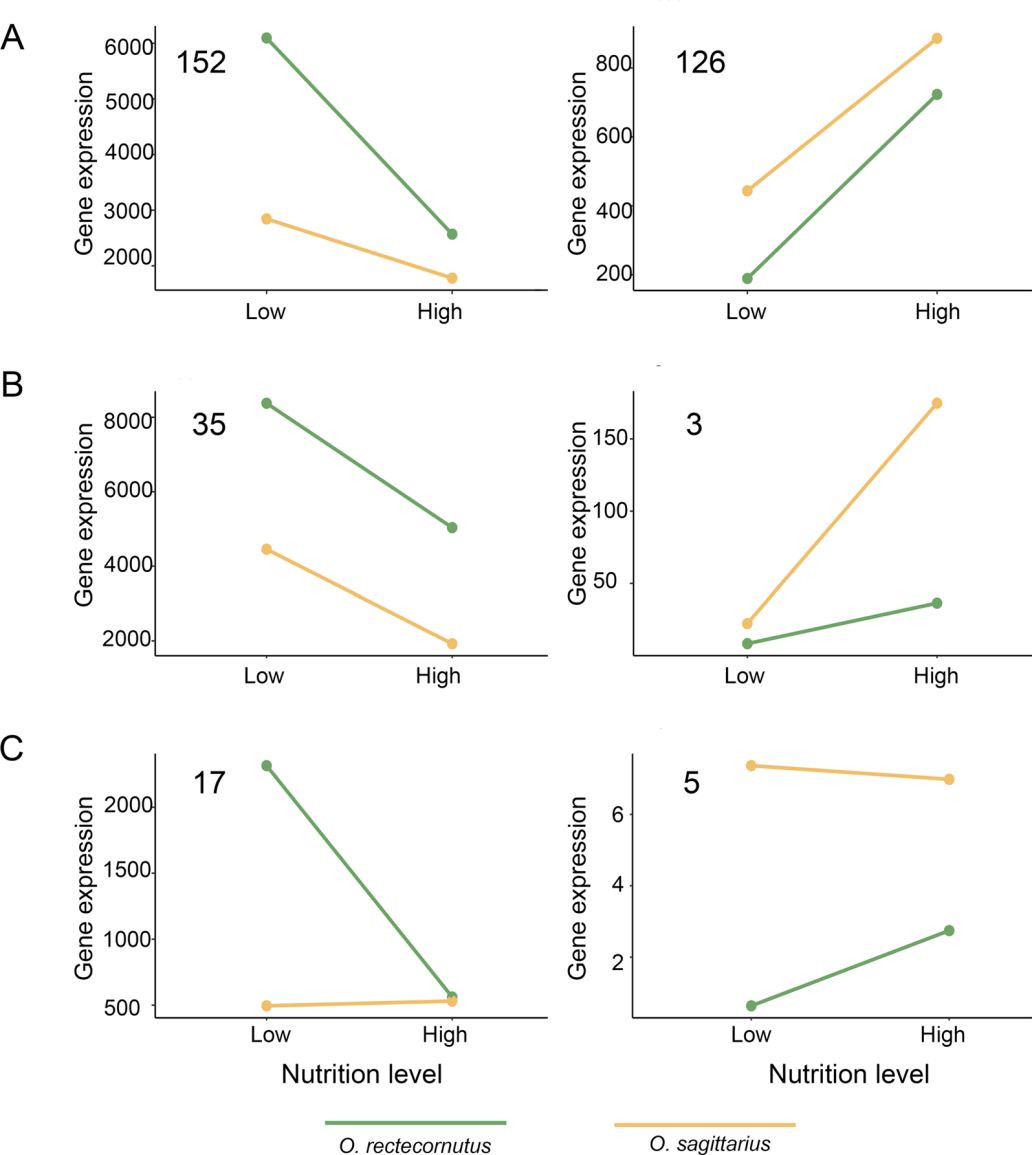
Differences in nutritional response patterns of the 338 shared NRGs between *O. rectecornutus* and *O. sagittarius*. A: 287 NRGs exhibited a stronger nutrition-responsive in *O. rectecornutus* than *O. sagittarius*. B: 38 NRGs displayed a stronger response in *O. sagittarius* than *O. rectecornutus*. C: 22 NRGs showed opposing response patterns between the two species. The log₂FC values between low- and high-nutrition conditions were calculated for each gene to determine direction of nutritional response, and genes were subsequently classified into different categories. Line plots represent average expression profiles of all genes within each defined category

To test whether gene-expression changes mirrored the direction and extent of morphological plasticity, we analyzed the 338 shared NRGs overlapped between *O. sagittarius* and *O. rectecornutus*, both of which possess enlarged female horns compared with the rudimentary horns of ancestral *O. bivertex*. Among these, 316 (H₀: p = 0.5, *P* < 0.001) genes showed concordant regulation in both species (Fig. 3A, B), whereas only 22 (H₀: p = 0.5, *P* < 0.001) showed opposing shifts (Fig. 3C; Supplementary Table S2). Moreover, a large proportion of genes (82.25%, 278/338) (H₀: p = 0.33, *P* < 0.001) exhibited a larger expression difference between low- and high-nutrition conditions in *O. rectecornutus* (Fig. 3A; Supplementary Table S2), which shows strong horn plasticity, 11.24% (38/338, H₀: p = 0.33, *P* < 0.001) were more nutrition-sensitive in *O. sagittarius* (Fig. 3B; Supplementary Table S2). A representative example is *Hh*, which has previously been implicated as a nutritional regulator mediating strong sensitivity to nutrition-dependent head horn growth in the bull-headed dung beetle *O. taurus* [34]. In our analysis, *Hh* was significantly upregulated in *O. rectecornutus,* which exhibits strong nutritional sensitivity of head horn growth, showed a modest upward trend under high-nutrition condition in *O. sagittarius*, and remained unchanged in *O. bivertex*, where head horns are largely insensitive to nutritional variation (Supplementary Figure S4). These findings suggest that the transcriptional architecture of horn plasticity evolves through both coordinated directional regulation of gene expression and lineage-specific differences in the magnitude of nutritional sensitivity.

### Enhancing nutritional plasticity of female horns primarily evolved through recruitment of conserved genes

TRGs are genes with no recognizable homologs outside a clade, frequently originating de novo or through rapid divergence [54, 55]. They are increasingly recognized as drivers of evolutionary innovation, contributing to ecological adaptation, morphological novelty, and lineage-specific diversification [54, 56–59]. However, a recent study indicated that TRGs contribute little to the developmental or evolutionary modulation of nutrition-responsive male horn plasticity, which instead primarily involved changes in the responsiveness of conserved genes [17]. To test whether this pattern applies to female horn plasticity, we identified TRGs and assessed nutritional responsiveness of both TRGs and conserved genes. 1427 TRGs in *O. rectecornutus* and 454 TRGs in *O. sagittarius* lacking identifiable orthologs in other species were identified. In *O. rectecornutus*, only 1.05% (15/1427) of TRGs were differentially expressed, compared with 4.74% (828/17491) of conserved genes (Supplementary Table S4). A similar trend was observed in *O. sagittarius*, 1.98% (9/454) of TRGs versus 7.88% (1285/16316) of conserved genes were differentially expressed. These findings suggest that the evolutionary enhancement of female head horn plasticity in derived species is largely mediated by the recruitment of conserved regulatory pathways rather than lineage-specific novelties, which is in line with the evolution of horn plasticity in males.

### Sexual differences in the enhancement of nutritional plasticity

**Fig. 4:**
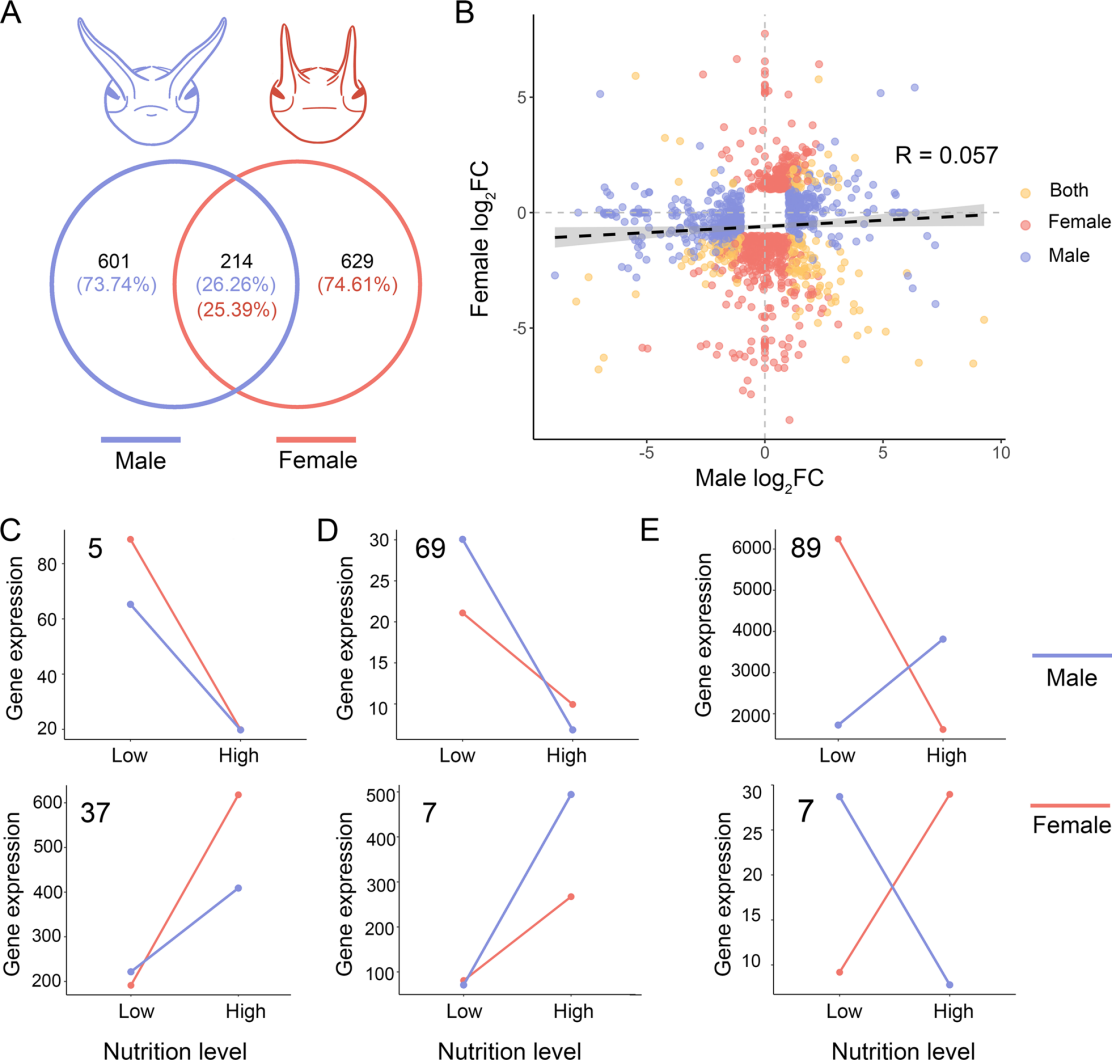
Sex-specific strategies in shaping nutritional plasticity of NRGs in *O. rectecornutus*. A: Distribution of NRGs classified by sex-specific or shared expression. B: Log₂FC values in response to nutrition for NRGs specific to males (blue), females (red), or shared between sexes (yellow). C: 42 NRGs exhibited a stronger response in female than male. D: 76 NRGs exhibited a stronger nutritional response in male than female. E: 96 NRGs showed opposing response patterns between the sexes, where an increase in one sex corresponds to a decrease in the other. In panels C and D, genes on the upper portion of the graph display a decreasing trend with improving nutrition, while those on the lower portion show an increasing trend. For C–E, log₂FC values between low- and high-nutrition conditions were calculated for each gene to determine direction of nutritional response, and genes were subsequently classified into different categories. Line plots represent average expression profiles of all genes within each defined category

Whether exaggerated traits in both sexes evolved through shared or distinct genetic mechanisms remains largely unresolved. To test this, we compared transcriptomic response of horn primordia to nutritional changes between male and female *O. rectecornutus*, in which both sexes possess exaggerated head horns. Differential expression analysis revealed pronounced sex bias, with 815 NRGs in males and 843 in females, and only 214 (Fisher’s exact test, *P* < 0.001) genes shared between the sexes (Fig. 4A). Expression correlation across sexes was low (R = 0.057) (Fig. 4B), underscoring largely independent nutritional responses. Among the shared NRGs, 118 genes displayed concordant responses to high- versus low-nutrition conditions in both sexes (H₀: p = 0.5, *P* = 0.151), with 42 showing stronger expression changes in females and 76 in males (Fig. 4C, D; Supplementary Table S3). In contrast, 96 shared NRGs exhibited sex-opposite nutritional responses (H₀: p = 0.33, *P* < 0.001), being upregulated under high nutrition in one sex and downregulated in the other (Fig. 4E; Supplementary Table S3), indicating substantially divergent regulation even when the same repertoires were recruited.

**Fig. 5:**
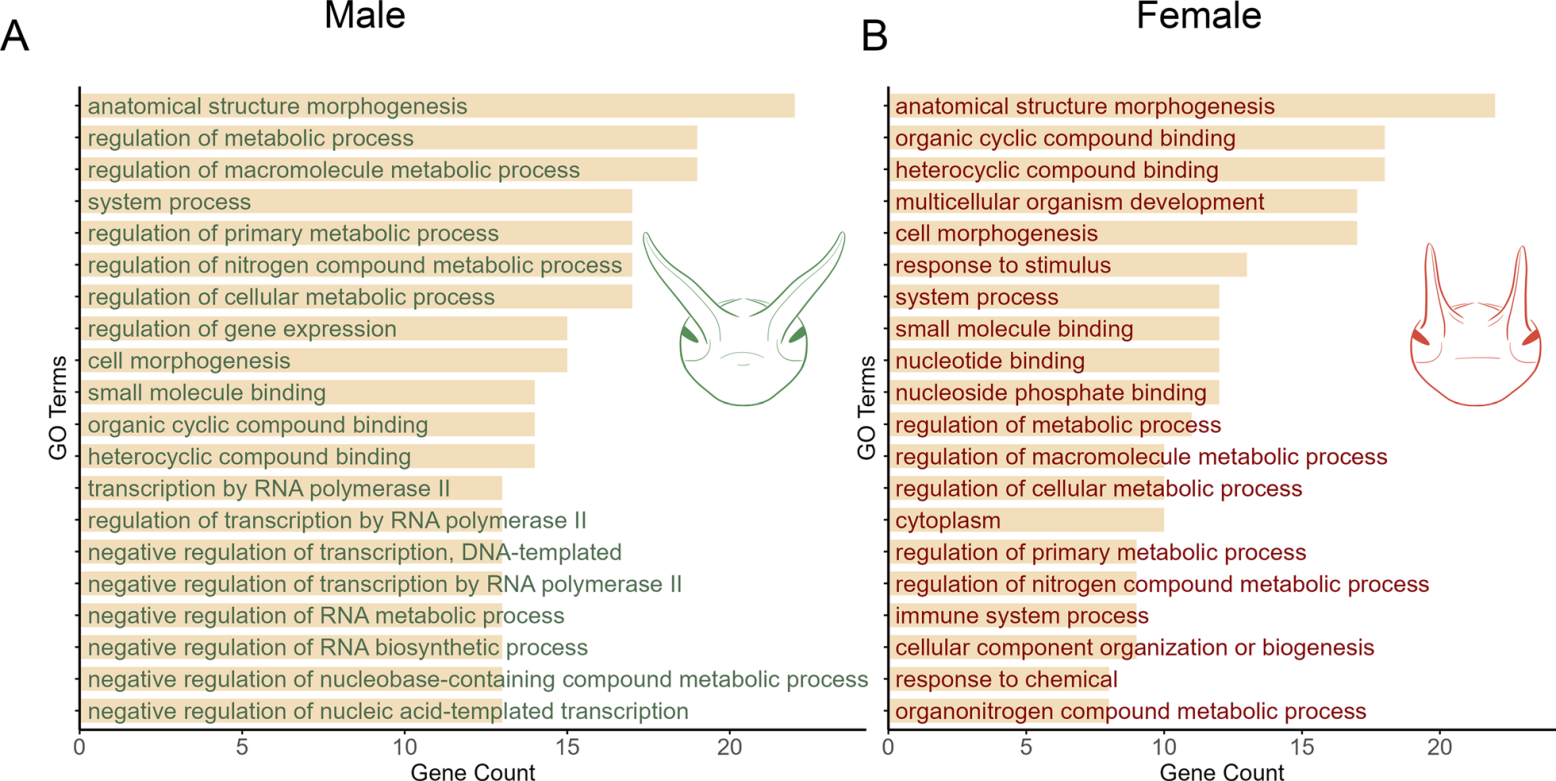
Gene Ontology (GO) enrichment analysis reveals functional specialization of sex-specific NRGs. A: Enriched functions in male-specific NRGs. B: Enriched functions in female-specific NRGs

GO enrichment further highlighted sex-specific biases. Male-specific NRGs were enriched in metabolic process regulation and gene expression control, whereas female-specific NRGs were enriched in pathways related to developmental and physiological adaptation (Fig. 5). Notably, several GO-terms, including anatomical structure morphogenesis, cell morphogenesis, and small molecule binding, were significantly enriched in both male- and female-specific NRGs. However, the gene sets underlying these enrichments were entirely non-overlapping between the sexes. This suggests that the apparent similarity at the GO-term level reflects convergence on broad developmental processes, while the transcriptional implementation of these processes is sex-specific. In contrast, shared NRGs between sexes were enriched in highly conserved biological pathways using KEGG enrichment analysis, including amino acid metabolism (tyrosine, glycine, serine, threonine, arginine, histidine), steroid and insect hormone biosynthesis, Hh signaling, and growth-regulatory pathways such as stem cell pluripotency and transcription factor networks (Supplementary Figure S5).

Additionally, TRGs activation showed marked sexual asymmetry: females recruited 1.05% of their TRGs in response to nutritional variation, approximately three times higher than the 0.35% observed in males (Supplementary Table S5). Together, these results demonstrate that both sexes exhibit nutritional plasticity but largely rely on distinct transcriptional architectures, pointing to sex-specific evolutionary trajectories in the elaboration of exaggerated horns.

## Discussion

Beetle horns in dung beetles exhibit pronounced nutritional plasticity, rendering them highly responsive to larval diet [3, 60]. While the developmental mechanisms underlying plastic development of male horns have been extensively studied [3, 29, 32, 34, 60–64], the evolution and regulation of female horn plasticity remain largely unexplored, despite striking interspecific variation. By focusing our analyses on transcriptional responses within the horn primordia, our results are interpreted as describing nutrition-responsive gene expression within the horn primordia, rather than systemic nutritional signaling processes operating at the whole-body level. Below we discuss the key implications of our findings.

First, enhanced female horn plasticity in *Onthophagus* beetles appears to have evolved primarily through the recruitment and modulation of conserved regulatory networks, with limited contributions from TRGs. Previous studies showed that conserved pathways such as insulin/IGFs and Hedgehog signaling underlie threshold responses to nutrition and morph transitions in males [17, 32, 64]. Our results extend this framework to females, where conserved genes were overrepresented among nutrition-responsive repertoires, whereas TRGs contributed minimally. This pattern indicates that evolutionary enhancement of female horn plasticity largely reflects the redeployment of pre-existing molecular circuits rather than the emergence of novel genetic elements. These findings are consistent with a broader evolutionary developmental biology (evo-devo) pattern in which most morphological innovations arise via modifications of conserved developmental modules [65–67]. Regulatory evolution—through changes in *cis*-regulatory sequences, spatiotemporal gene expression, or network connectivity—provides a flexible and low-cost route for generating novel traits [67]. In contrast, gene duplication or de novo gene birth, though capable of producing genuine novelty, is more stochastic and evolutionarily costly due to the constraints of sequence evolution and pleiotropy [68, 69].

Nonetheless, exceptions exist, demonstrating that TRGs can underpin lineage-restricted novelties in specific ecological contexts, such as parasitic wasp venoms or the propelling fan of *Rhagovelia* water striders [56, 70]. The contrast between these cases and the conserved-network recruitment observed in female *Onthophagus* horns underscores the diversity of molecular routes to morphological evolution. Our data suggest that enhancement of nutritional plasticity in female horns exemplifies morphological diversification predominantly mediated by regulatory evolution acting on the expression of conserved gene networks—a mechanism that balances evolvability with developmental stability, which not excluding the involvement of a small number of functionally important TRG-associated DEGs. Thus, functional evaluation such as RNA interference will be necessary to evaluate the contributions of conserved genes versus TRGs in the future study.

Second, the transcriptional repertoires underlying nutrition-sensitive horn development are largely lineage-specific, suggesting repeated, independent co-option of gene networks during evolutionary transitions in plasticity. The recruitment of a shared set of genes in *O. sagittarius* and *O. rectecornutus* suggests that conserved plastic programs can be repeatedly co-opted to generate novel traits, while lineage-specific shifts illustrate how independent solutions can converge on superficially similar outcomes. This pattern mirrors broader principles in evo-devo, where repeated origins of complex traits often involve parallel redeployment of conserved networks coupled with lineage-specific divergence [57, 71–75].

Third, based on classical morphological and developmental studies in dung beetles [24, 76, 77], as well as our own observations, the head horns located in the dorsal-posterior region of the head in both males and females are generally considered as homologous structures, arising from the same anatomical region during development. Despite this shared developmental origin, comparisons between sexes in *O. rectecornutus* reveal that their transcriptional responses to nutrition are largely decoupled. Only 214 of the NRGs were shared, and nearly half of them exhibited opposing regulation between sexes. Such transcriptional divergence, despite the shared presence of homologous horns, suggests that males and females achieve horn exaggeration through distinct genetic architectures, consistent with evidence that the downstream repertoires of Dsx*,* a conserved sex-determination regulator, are highly sex-specific [33, 78]. Importantly, these findings align with the contrasting selective contexts in which horns evolve: in males as sexually selected weapons mediating competition for mates [3, 61]; in females as ecologically selected weapons mediating contests over breeding resources [79]. The transcriptional divergence documented here likely reflects these sex-specific selective regimes. Similar patterns of sex-biased plasticity have been documented in immunity [80], metabolism [81], and secondary sexual traits in birds and insects [82], suggesting that sex-specific regulation is probably a general principle governing not only sexual dimorphism but also evolution of plastic and exaggerated traits.

Finally, we note that our conclusions are based on transcriptomic analyses of horn primordia rather than direct functional assays. While these results identified genes and pathways responsive to nutritional conditions, experimental validation will be necessary to confirm their roles in horn plastic development. Nonetheless, our analyses provide a set of candidate genes and regulatory networks to guide future functional studies of lineage- and sex-specific horn plasticity.

## Conclusions

Taken together, our findings indicate that female horn plasticity evolved through repeated co-option and rewiring of conserved developmental networks, coupled with lineage-specific transcriptional divergence. These results highlight the distinct developmental basis and evolutionary trajectories of male and female horns, and more broadly, they underscore how conserved genetic toolkits can be differentially mobilized by sex- and lineage-specific selection to drive the evolution of novel, plastic, and exaggerated traits.

## Supplementary Information

Additional file1 (DOCX 1304 KB)

## Data Availability

The transcriptome data generated in this study have been deposited in the NCBI database (PRJNA1358597). All data generated or analyzed during this study are included in this published article and its supplementary files.
