# Supplementary material for: Transcriptomic basis underlying the evolution of female horn plasticity in scarab beetles
Source: Front Zool. 2026 Feb 5;23:6. doi: 10.1186/s12983-026-00597-3 (PMC12973629; doi:10.1186/s12983-026-00597-3)
Supplement: Supplementary file 1 — Additional file1 (DOCX 1304 KB) [file 12983_2026_597_MOESM1_ESM.docx]

**Supplementary material**


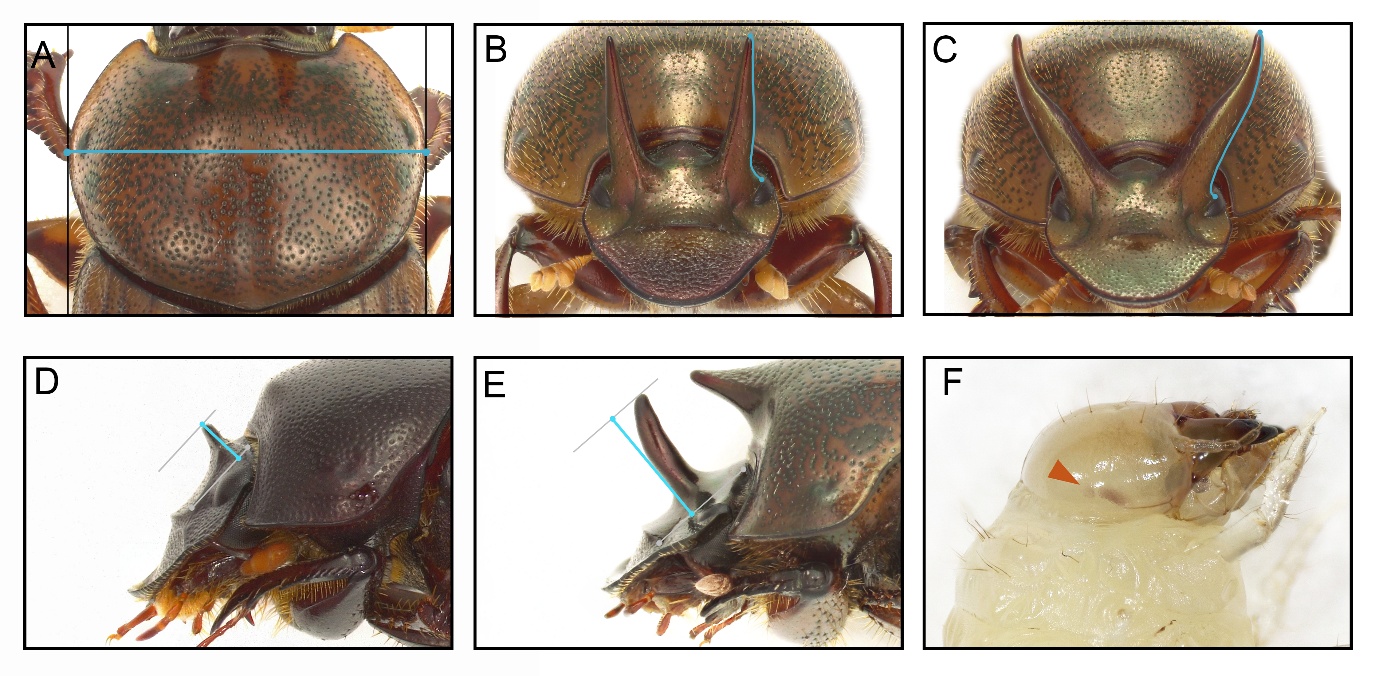


Supplementary Figure S1 Morphometric criteria for assessing scaling relationships. A: Measurement of prothoracic width (blue line). B-E: Measurements of horn length (blue lines) for male *O. rectecornutus* (B), female *O. rectecornutus* (C), female *O. bivertex* (D), and female *O. sagittarius* (E). F: Late prepupa stage. The position of compound eye was indicated with arrowhead.


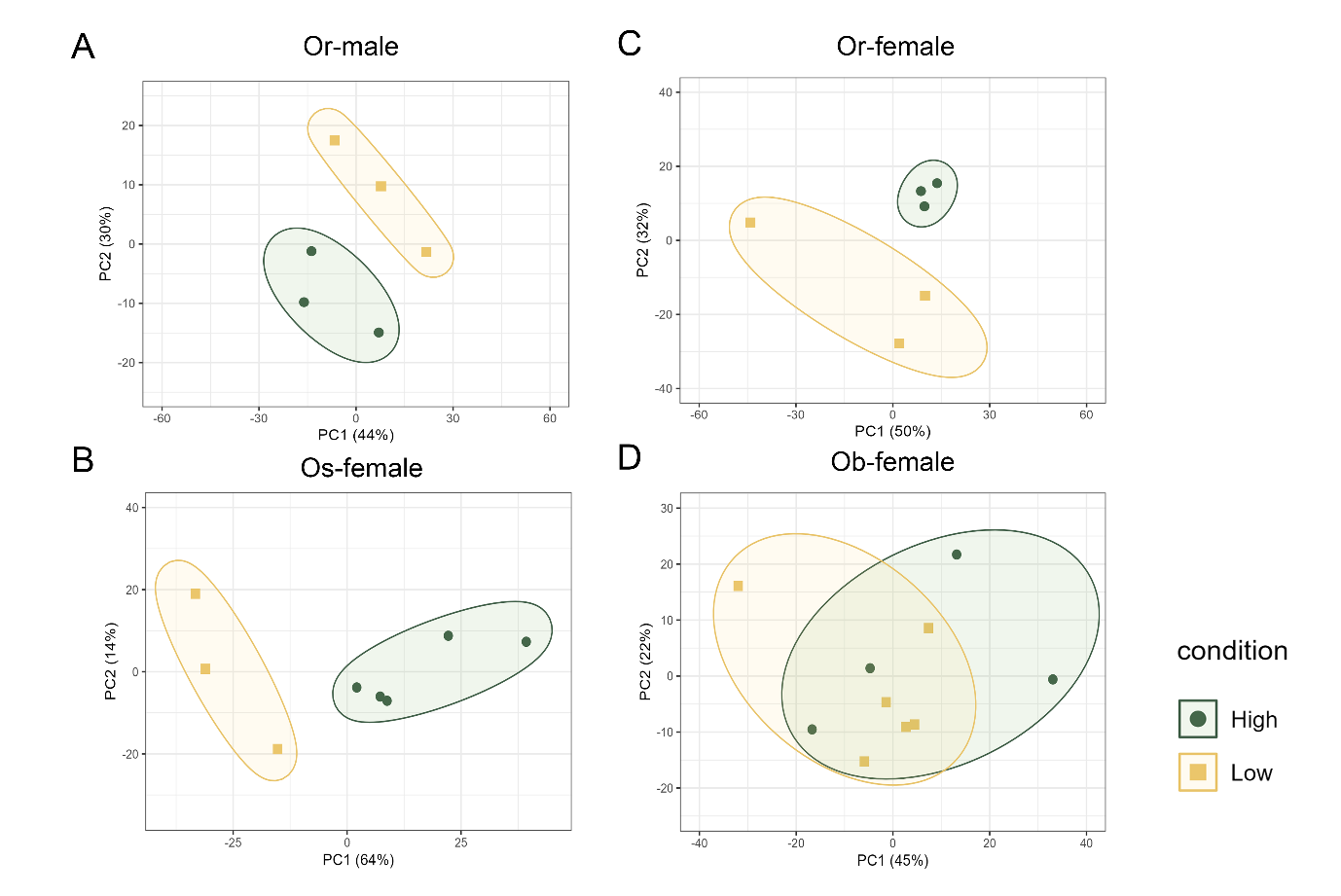


Supplementary Figure S2 Principal component analysis (PCA) of phenotypic responses of three *Onthophagus* species under low- (yellow) and high-nutritions (green). A: male *O. rectecornutus*. B: female *O. rectecornutus.* C: female *O. sagittarius.* D: female *O. bivertex.*


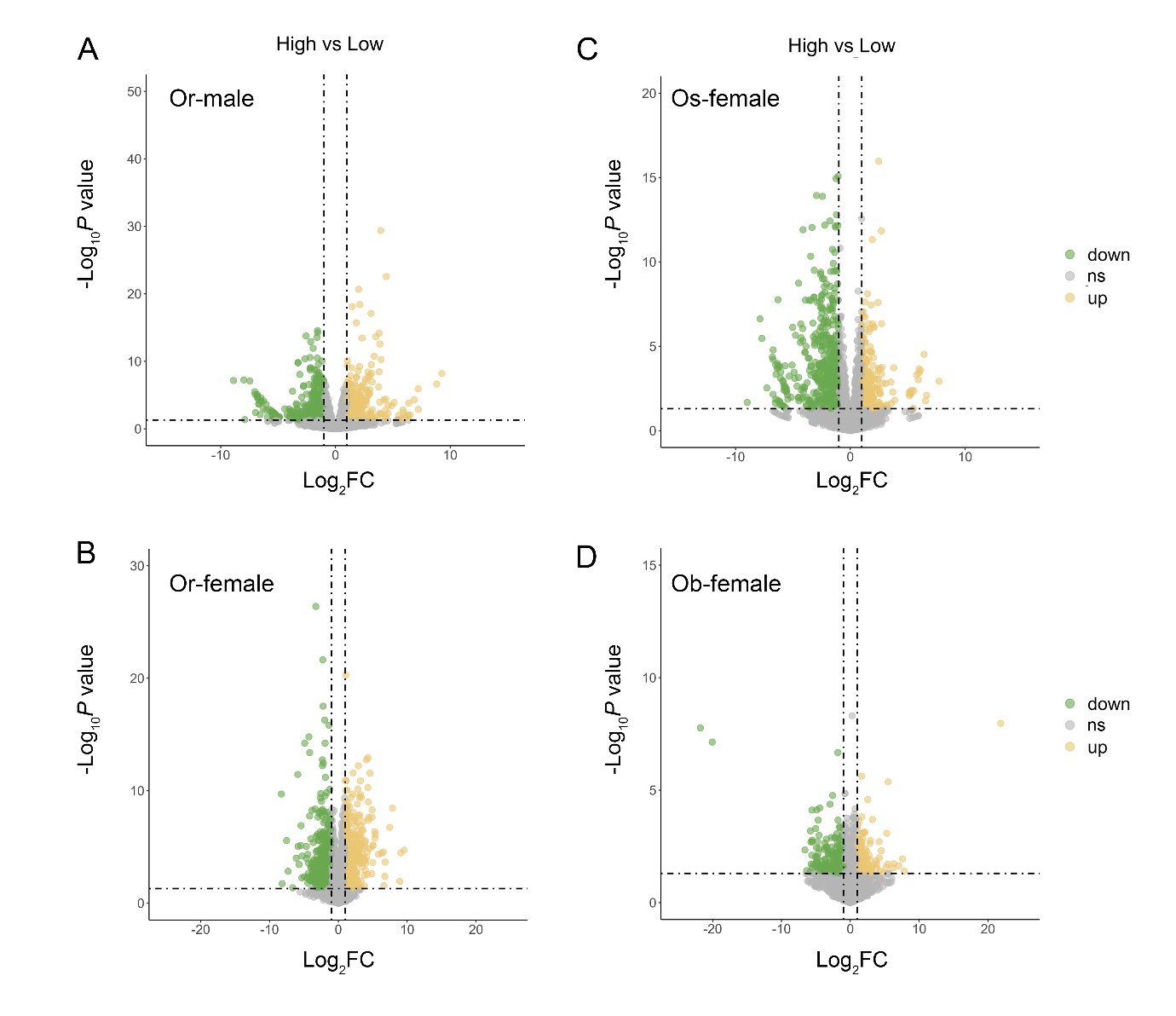


Supplementary Figure S3 Volcano plots of NRG expression changes under high- versus low-nutritions. A: Male *O. rectecornutus*. B: Female *O. rectecornutus.* C: Female *O. sagittarius.* D: Female *O. bivertex.* Significant NRGs (|log_2_FC| > 1, *P* < 0.05) are highlighted in yellow (up-regulation) and green (down-regulation). Grey: not significant (ns)


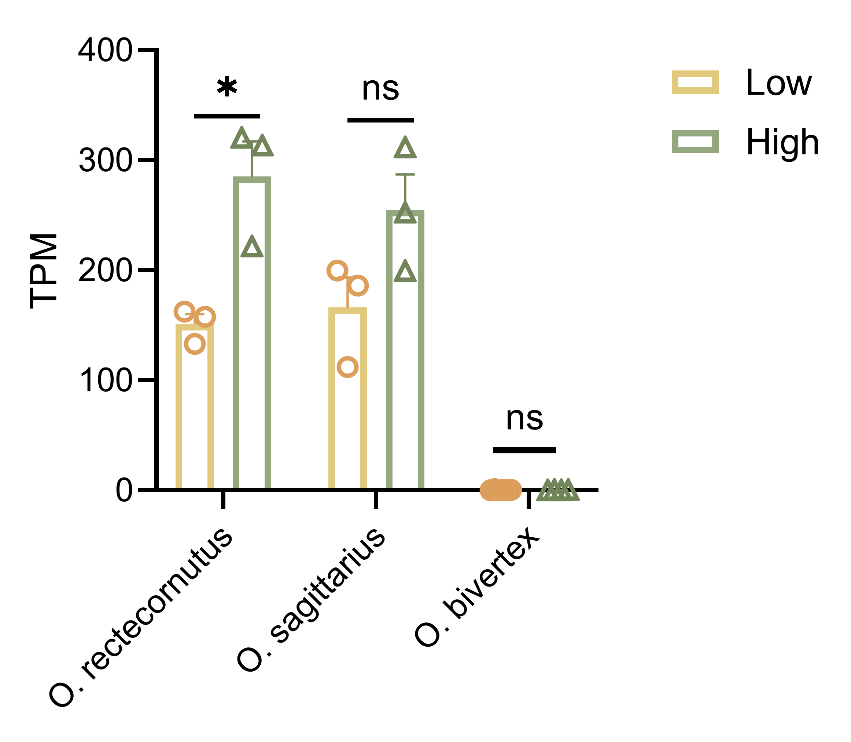


Supplementary Figure S4 Relative expression levels of *Hh* under low- (yellow) and high-nutrition conditions (green) in the horn primordia of three *Onthophagus* species. The data are presented as the average TPM  ±  SEM (n  ≥ 3). Statistical significance was assessed using a two-tailed Student’s *t*-test (*P* < 0.05).


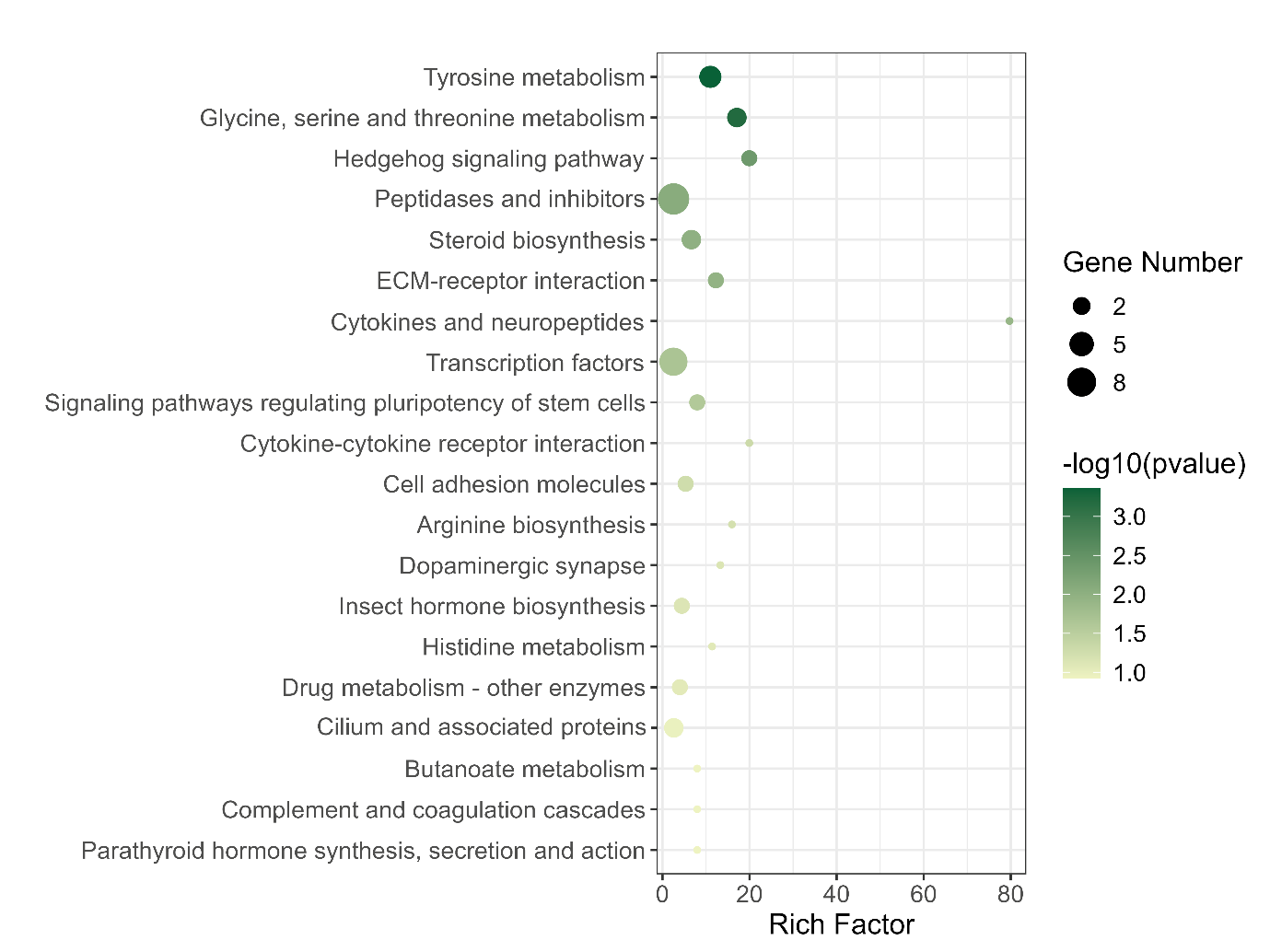


Supplementary Figure S5 KEGG enrichment analysis reveals functional specialization of sex-shared NRGs. Circle size indicates the number of genes, and color intensity represents the enrichment level (−log₁₀ *P* value).

Supplementary Table S1 CVs of three gene sets in *O. rectecornutus*, *O. sagittarius*, and *O. bivertex*.

Pairwise comparisons were performed using Welch’s t-tests and Bonferroni correction.

| Gene sets | Group | Category | Comparison | t | *P adj* | Significant | Figure |
| --- | --- | --- | --- | --- | --- | --- | --- |
| Moderate NRGs | NRGs: 461 | *O. sagittarius* | High vs Low | -3.64508 | 0.001148953 | * | Fig.2C |
|  |  | *O. bivertex* | High vs Low | -0.53772 | 1 |  |  |
|  |  | High-nutrition | Os vs Ob | 4.20593 | 0.000126145 | * |  |
|  |  | Low-nutrition | Os vs Ob | 5.8458 | 3.94E-08 | * |  |
|  | NRGs: 252 | *O. sagittarius* | High vs Low | 2.54285 | 0.29283425 |  |  |
|  |  | *O. bivertex* | High vs Low | 0.032643 | 1 |  |  |
|  |  | High-nutrition | Os vs Ob | 0.38559 | 1 |  |  |
|  |  | Low-nutrition | Os vs Ob | -0.087217 | 1 |  |  |
| Strong NRGs | NRGs: 230 | *O. sagittarius* | High vs Low | 1.18747 | 0.944148878 |  | Fig.2D |
|  |  | *O. bivertex* | High vs Low | -0.88869 | 1 |  |  |
|  |  | High-nutrition | Or vs Ob | 3.4433 | 0.002708172 | * |  |
|  |  | Low-nutrition | Or vs Ob | 2.85598 | 0.018681991 | * |  |
|  | NRGs: 104 | *O. sagittarius* | High vs Low | 3.671495 | 0.001496608 | * |  |
|  |  | *O. bivertex* | High vs Low | 0.091612 | 1 |  |  |
|  |  | High-nutrition | Or vs Ob | 4.33284 | 0.000148352 | * |  |
|  |  | Low-nutrition | Or vs Ob | 3.17106 | 0.00796017 | * |  |
| Shared NRGs | NRGs: 187 | *O.rectecornutus* | High vs Low | 3.121985 | 0.017849198 | * | Fig.2E |
|  |  | *O. sagittarius* | High vs Low | -2.63877 | 0.079113352 |  |  |
|  |  | *O. bivertex* | High vs Low | 0.567697 | 1 |  |  |
|  |  | High-nutrition | Or vs Ob | 3.42626 | 0.006750151 | * |  |
|  |  | Low-nutrition | Or vs Ob | 3.59033 | 0.00380022 | * |  |
|  |  | High-nutrition | Os vs Ob | 1.31154 | 1 |  |  |
|  |  | Low-nutrition | Os vs Ob | 5.10743 | 7.54E-06 | * |  |
|  |  | High-nutrition | Or vs Os | -3.52523 | 0.00447882 | * |  |
|  |  | Low-nutrition | Or vs Os | 2.45957 | 0.130603493 |  |  |
|  | NRGs: 129 | Or | High vs Low | 9.448026 | 1.92E-15 | * |  |
|  |  | Os | High vs Low | 4.503351 | 0.000118278 | * |  |
|  |  | Ob | High vs Low | -0.18129 | 1 |  |  |
|  |  | High-nutrition | Or vs Ob | 4.00278 | 0.001160539 | * |  |
|  |  | Low-nutrition | Or vs Ob | 0.66527 | 1 |  |  |
|  |  | High-nutrition | Os vs Ob | 1.23172 | 1 |  |  |
|  |  | Low-nutrition | Os vs Ob | 0.92571 | 1 |  |  |
|  |  | High-nutrition | Or vs Os | -5.30512 | 3.28E-06 | * |  |
|  |  | Low-nutrition | Or vs Os | 0.217137 | 1 |  |  |

Supplementary Table S2 CVs of the shared NRGs in *O. rectecornutus* and *O. sagittarius*.

Pairwise comparisons were performed using Welch’s t-tests and Bonferroni correction.

| Gene set | Group | Category | Comparison | t | *P adj* | Significant | Figure |
| --- | --- | --- | --- | --- | --- | --- | --- |
| Shared NRGs | NRGs: 152 | *O. rectecornutus* | High vs Low | 3.370355 | 3.40E-03 | * | Fig.3A |
|  |  | *O. sagittarius* | High vs Low | -3.72352 | 0.000938798 | * |  |
|  |  | High-nutrition | Or vs Os | 2.73145 | 0.026844192 | * |  |
|  |  | Low-nutrition | Or vs Os | -4.298138 | 9.39E-05 | * |  |
|  | NRGs: 126 | *O. rectecornutus* | High vs Low | 11.26799 | 1.18E-21 | * |  |
|  |  | *O. sagittarius* | High vs Low | 5.552887 | 3.36E-07 | * |  |
|  |  | High-nutrition | Or vs Os | 5.94923 | 4.37E-08 | * |  |
|  |  | Low-nutrition | Or vs Os | -0.571957 | 1 |  |  |
|  | NRGs: 35 | *O. rectecornutus* | High vs Low | 1.048166 | 1 |  | Fig.3B |
|  |  | *O. sagittarius* | High vs Low | -1.94735 | 0.222888169 |  |  |
|  |  | High-nutrition | Or vs Os | 4.73891 | 4.93E-05 | * |  |
|  |  | Low-nutrition | Or vs Os | 1.37251 | 0.699551085 |  |  |
|  | NRGs: 3 | *O. rectecornutus* | High vs Low | 0.635259 | 1 |  |  |
|  |  | *O. sagittarius* | High vs Low | 0.183818 | 1 |  |  |
|  |  | High-nutrition | Or vs Os | 2.03872 | 0.447558877 |  |  |
|  |  | Low-nutrition | Or vs Os | 1.05289 | 1 |  |  |
|  | NRGs: 17 | *O. rectecornutus* | High vs Low | -0.11527 | 1 |  | Fig.3C |
|  |  | *O. sagittarius* | High vs Low | 1.030534 | 1 |  |  |
|  |  | High-nutrition | Or vs Os | -0.112113 | 1 |  |  |
|  |  | Low-nutrition | Or vs Os | 1.02625 | 1 |  |  |
|  | NRGs: 5 | *O. rectecornutus* | High vs Low | 2.041805 | 0.444693537 |  |  |
|  |  | *O. sagittarius* | High vs Low | 0.759899 | 1 |  |  |
|  |  | High-nutrition | Or vs Os | 0.99034 | 1 |  |  |
|  |  | Low-nutrition | Or vs Os | -0.994594 | 1 |  |  |

Supplementary Table S3 CV of the shared NRGs between male and *female in O. rectecornutus*.

Pairwise comparisons were performed using Welch’s t-tests and Bonferroni correction.

| Gene set | Group | Category | Comparison | Sexes | t | *P adj* | Significant | Figure |
| --- | --- | --- | --- | --- | --- | --- | --- | --- |
| Sex-shared NRGs | NRGs: 5 | *O. rectecornutus* | High vs Low | Female | 1.672242286 | 0.729290881 |  | Fig. 4C |
|  |  | *O. rectecornutus* | High vs Low | Male | -1.732635997 | 0.588704155 |  |  |
|  |  | High-nutrition | Male vs Female |  | -0.812108037 | 1 |  |  |
|  |  | Low-nutrition | Male vs Female |  | 2.115954601 | 0.460399704 |  |  |
|  | NRGs: 37 | *O. rectecornutus* | High vs Low | Female | 3.642020106 | 0.002504377 | * |  |
|  |  | *O. rectecornutus* | High vs Low | Male | 1.009609009 | 1 |  |  |
|  |  | High-nutrition | Male vs Female |  | -2.110497733 | 0.157522425 |  |  |
|  |  | Low-nutrition | Male vs Female |  | 0.581502343 | 1 |  |  |
|  | NRGs: 69 | *O. rectecornutus* | High vs Low | Female | 4.003201187 | 0.000488653 | * | Fig. 4D |
|  |  | *O. rectecornutus* | High vs Low | Male | -0.206893873 | 1 |  |  |
|  |  | High-nutrition | Male vs Female |  | -2.779362944 | 0.02687052 | * |  |
|  |  | Low-nutrition | Male vs Female |  | 1.053500486 | 1 |  |  |
|  | NRGs: 7 | *O. rectecornutus* | High vs Low | Female | 0.196540645 | 1 |  |  |
|  |  | *O. rectecornutus* | High vs Low | Male | 0.827005094 | 1 |  |  |
|  |  | High-nutrition | Male vs Female |  | 1.078085938 | 1 |  |  |
|  |  | Low-nutrition | Male vs Female |  | 0.43583583 | 1 |  |  |
|  | NRGs: 89 | *O. rectecornutus* | High vs Low | Female | 0.627817796 | 1 |  | Fig. 4E |
|  |  | *O. rectecornutus* | High vs Low | Male | -1.043892461 | 1 |  |  |
|  |  | High-nutrition | Male vs Female |  | -0.572401207 | 1 |  |  |
|  |  | Low-nutrition | Male vs Female |  | 1.278964345 | 0.812021219 |  |  |
|  | NRGs: 7 | *O. rectecornutus* | High vs Low | Female | 1.354702227 | 1 |  |  |
|  |  | *O. rectecornutus* | High vs Low | Male | 1.193174181 | 1 |  |  |
|  |  | High-nutrition | Male vs Female |  | -1.474712011 | 0.865062877 |  |  |
|  |  | Low-nutrition | Male vs Female |  | -0.136026304 | 1 |  |  |

Supplementary Table S4 Proportions of TRGs and conserved genes in *O. rectecornutus* and *O. sagittarius*.

| Species | Category | NRGs | Nutrition-irresponsive genes | Total |
| --- | --- | --- | --- | --- |
| *O. rectecornutus* | TRGs | 15 (1.05%) | 1412 (98.95%) | 1427 (100%) |
|  | Conserved genes | 828 (4.74%) | 16663 (95.26%) | 17491 (100%) |
| *O. sagittarius* | TRGs | 9 (1.98%) | 445 (98.02%） | 454 (100%) |
|  | Conserved genes | 1285 (7.88%) | 15031 (92.12%) | 16316 (100%) |

Supplementary Table S5 Proportions of TRGs and conserved genes within female and male.

| Sexes | Category | NRGs | Nutrition-irresponsive genes | Total |
| --- | --- | --- | --- | --- |
| Male | TRGs | 5 (0.35%) | 1422 (99.65%) | 1427 (100%) |
|  | Conserved genes | 810 (4.63%) | 16 681 (95.40%) | 17491 (100%) |
| Female | TRGs | 15 (1.05%) | 1412 (98.95%) | 1427 (100%) |
|  | Conserved genes | 828 (4.73%) | 16663 (95.27%) | 17491 (100%) |

Supplementary Table S6 Quality assessment of RNA samples for transcriptome sequencing

| Sample name | Species | Treatment | Concentration (µg/µL) | OD  260/280 | OD  260/230 | RIN |
| --- | --- | --- | --- | --- | --- | --- |
| Ob-L-female1 | *O. bivertex* | Low-nutrition | 0.17 | 2.12 | 2.20 | 10.0 |
| Ob-L-female2 | *O. bivertex* | Low-nutrition | 0.13 | 2.10 | 1.99 | 10.0 |
| Ob-L-female3 | *O. bivertex* | Low-nutrition | 0.15 | 2.15 | 2.10 | 9.9 |
| Ob-L-female4 | *O. bivertex* | Low-nutrition | 0.09 | 2.09 | 1.82 | 9.7 |
| Ob-L-female5 | *O. bivertex* | Low-nutrition | 0.14 | 2.03 | 1.75 | 9.6 |
| Ob-L-female6 | *O. bivertex* | Low-nutrition | 0.08 | 2.08 | 1.88 | 9.9 |
| Ob-H-female1 | *O. bivertex* | High-nutrition | 0.17 | 2.06 | 2.13 | 10.0 |
| Ob-H-female2 | *O. bivertex* | High-nutrition | 0.20 | 2.11 | 2.12 | 10.0 |
| Ob-H-female3 | *O. bivertex* | High-nutrition | 0.20 | 2.02 | 2.05 | 10.0 |
| Ob-H-female4 | *O. bivertex* | High-nutrition | 0.20 | 2.03 | 1.87 | 10.0 |
| Or-L-female1 | *O. rectecornutus* | Low-nutrition | 0.13 | 2.05 | 1.73 | 10.0 |
| Or-L-female2 | *O. rectecornutus* | Low-nutrition | 0.18 | 2.06 | 1.78 | 9.7 |
| Or-L-female3 | *O. rectecornutus* | Low-nutrition | 0.12 | 2.15 | 1.84 | 9.9 |
| Or-L-male1 | *O. rectecornutus* | Low-nutrition | 0.21 | 2.09 | 2.20 | 10.0 |
| Or-L-male2 | *O. rectecornutus* | Low-nutrition | 0.14 | 2.11 | 1.78 | 9.6 |
| Or-L-male3 | *O. rectecornutus* | Low-nutrition | 0.14 | 2.13 | 2.05 | 9.6 |
| Or-H-female1 | *O. rectecornutus* | High-nutrition | 0.34 | 2.09 | 1.97 | 10.0 |
| Or-H-female2 | *O. rectecornutus* | High-nutrition | 0.28 | 2.15 | 1.99 | 10.0 |
| Or-H-female3 | *O. rectecornutus* | High-nutrition | 0.09 | 1.95 | 1.78 | 10.0 |
| Or-H-male1 | *O. rectecornutus* | High-nutrition | 0.51 | 2.18 | 2.2 | 10.0 |
| Or-H-male2 | *O. rectecornutus* | High-nutrition | 0.28 | 2.12 | 2.19 | 10.0 |
| Or-H-male3 | *O. rectecornutus* | High-nutrition | 0.10 | 1.83 | 1.82 | 10.0 |
| Os-L-female1 | *O. sagittarius* | Low-nutrition | 0.19 | 2.08 | 2.20 | 10.0 |
| Os-L-female2 | *O. sagittarius* | Low-nutrition | 0.23 | 2.08 | 1.74 | 9.5 |
| Os-L-female3 | *O. sagittarius* | Low-nutrition | 0.18 | 2.09 | 2.18 | 9.6 |
| Os-H-female1 | *O. sagittarius* | High-nutrition | 0.13 | 2.08 | 2.07 | 10.0 |
| Os-H-female2 | *O. sagittarius* | High-nutrition | 0.30 | 2.02 | 2.06 | 10.0 |
| Os-H-female3 | *O. sagittarius* | High-nutrition | 0.34 | 2.09 | 2.02 | 10.0 |
| Os-H-female4 | *O. sagittarius* | High-nutrition | 0.17 | 2.04 | 1.76 | 9.7 |
| Os-H-female5 | *O. sagittarius* | High-nutrition | 0.12 | 2.08 | 1.88 | 9.8 |
